# Party package travel: alcohol use and related problems in a holiday resort: a mixed methods study

**DOI:** 10.1186/1471-2458-8-351

**Published:** 2008-10-07

**Authors:** Morten Hesse, Sébastien Tutenges, Sanna Schliewe, Tine Reinholdt

**Affiliations:** 1 Aarhus University, Centre for Alcohol and Drug Research, Copenhagen Division, Købmagergade 26E, 1150 Copenhagen C, Denmark

## Abstract

**Background:**

People travelling abroad tend to increase their use of alcohol and other drugs. In the present study we describe organized party activities in connection with young tourists' drinking, and the differences between young people travelling with and without organized party activities.

**Methods:**

We conducted ethnographic observations and a cross-sectional survey in Sunny Beach, Bulgaria.

**Results:**

The behaviour of the guides from two travel agencies strongly promoted heavy drinking, but discouraged illicit drug use. Even after controlling for several potential confounders, young people who travelled with such "party package travel agencies" were more likely to drink 12 or more units when going out. In univariate analyses, they were also more likely to get into fights, but were not more likely to seek medical assistance or medical assistance for an accident or an alcohol-related problem. After controlling for confounders, the association between type of travel agency and getting into fights was no longer significant. Short-term consequences of drinking in the holiday resort did not differ between party package travellers and ordinary package travellers.

**Conclusion:**

There may be a small impact of party package travels on young people's drinking. Strategies could be developed used to minimise the harm associated with both party package travel and other kinds of travel where heavy substance use is likely to occur.

## Background

Millions of young people travel abroad on holiday for one or two weeks each year. A large proportion of these young travellers are attracted to resorts with a wild party scene and easy access to intoxicants [1].

A number of adverse health consequences have been observed from substance use in nightlife, including fights, accidents, and a range of other negative effects [2]. Heavy drinking in particular may lead to negative consequences, such as blackouts, personal injuries, and sudden death. Heavy drinking may also contribute to accidents, violence and rape [3], and be a risk factor for sexually transmitted diseases [4] and the development of alcohol dependence [5]. On the other hand, drinking alcohol in the Nordic culture has strong links with the perception of maturity and adulthood, and with drinking heavily and losing control [6].

Substance use undertaken in a foreign country has additional risks: language and geography are generally unfamiliar and this may obstruct access to health services, and individuals far from home are not held back by the constraints of work and family that normally moderate substance use [7]. Accordingly, a range of studies have identified amplified substance use in people on holidays abroad [8-10]. Young people who come to destinations attracted by the "party reputation" of a scene consume even more drugs and alcohol than other young people [4].

As shown by Bellis et al. [11], patterns of substance use differ in travellers going on different kinds of travels. For instance, young British travellers in Ibiza have an unusually high consumption of drugs whereas young British backpackers in Australia have an increased use of alcohol rather than drugs. But how do travel agents affect the use of intoxicants among travellers? And how can travel agents improve their strategies to minimise substance use and the harms associated with substance use? In this paper these questions will be treated by a case study of young Danish travellers going to the Bulgarian nightlife resort Sunny Beach (SB).

During the summer 2007, approximately 5100 young Danes in the ages 16 to 30 years went to SB with party package agencies, according to information from the agencies. A probably similar but unknown number of young Danes travelled with traditional travel agencies. The Centre for Alcohol and Drug Research conducted a study of the drinking behaviour of young Danes in SB during this period.

## Methods

### Ethnographic fieldwork

This study draws on ethnographic fieldwork conducted by one if the authors at SB in the period from June 19 to August 12, 2007. Systematic observations were made in the daytime and nighttime at bars, discotheques, hotels, the beach and other locations where Danish tourists and guides could be found. The observations were made overtly. They were written down on paper in situ, for instance while sitting at a bar desk. Shortly after they were completed and typed on a computer. As a complement to the observations, semi structured tape-recorded interviews were carried out with Danish tourists and guides. One to six persons were interviewed at a time. In all, 27 interviews were tape recorded with a total of 63 people, 9 of whom were guides. In addition, we have scrutinized travel agents' web pages and made systematic assessments of the health problems in a selection of venues in SB.

### Cross-sectional survey

A cross-sectional survey was undertaken at Bourgas airport between July 4^th ^and July 21, 2007. Tourists holidaying in SB mainly use this airport. The procedure and questionnaire were highly similar to the procedure used in studies at Ibiza by Bellis and colleagues [10]. Details of the survey study have previously been published [12].

Research assistants were instructed to target young people, approximately 16–30 years old, and approach them while they were waiting to check in for their return flights to Denmark.

Only individuals returning to Denmark were asked to complete the short, anonymous questionnaire. Data collected included individuals' basic demographics and levels of substance use (alcohol, tobacco, illicit drugs) in Denmark and during the current holiday in SB. Individuals were also asked whether their parents were staying at SB, and which travel agency they were travelling with.

We collected questionnaires on 13 different days at a total of 30 flights. We strived to approach as many young people as possible at each flight. Researchers approached potential participants and asked if they had time to fill in a short questionnaire for a research project conducted by Aarhus University. Gender of those who refused was recorded. Consenting participants were then informed of the nature of the questionnaire, and if they refused at this stage, their gender was recorded.

Data collected included individuals' basic demographics, and levels of substance use (alcohol, tobacco, amphetamine, ketamine, cannabis, ecstasy, LSD, cocaine and GHB) in Denmark and during the current holiday in SB. The questionnaire was similar in layout to the questionnaire used in the Ibiza surveys, [10], but due to the different nature of the party scene in SB, specific questions about ecstasy use were replaced by questions about binge drinking.

The questionnaire also contained true false items related to getting into fights, seeking medical assistance, and number of sexual partners. After the item about medical assistance ("Have you had to go to hospital or see a doctor whilst on this holiday"), the next part was labelled "If yes, what was this for?" This was followed by four boxes. These boxes were labelled "Drug related problem or accident", "Alcohol related problem or accident", "Other accident" and "Illness". In the following, we refer to seeking medical assistance as those who said yes to the item about medical assistance, and as seeking medical assistance for an alcohol related problem those who said yes to both the medical assistance item and the alcohol related problem.

The questionnaire contained questions about frequency of drinking broken into the following categories: drinking more than 6 units per day, drinking more than 12 units per day, and drinking to intoxication. Note that due to time restrictions of youth waiting to check in on a flight, we had limited time and space to explain concepts such as "alcohol unit". Therefore, the respondents' understanding of a unit may have varied. Moreover, drink sizes and contents vary substantially in the venues of SB. Some venues serve drinks with large amounts of alcohol, others serve drinks with low amounts of alcohol – it is almost impossible to taste the difference. Therefore, there is some uncertainty about the actual amount consumed.

### Analyses of the survey data

Analysis utilised a combination of χ^2^, Spearman Rank order correlations, and ordinal regression analysis. As the main indicator of drinking, we used the proportion of days in SB, where subjects reported drinking 12 or more units of alcohol (number of days/days of drinking>12 units). This variable was recoded into three levels: Never, one to five days per week during the vacation, and six to seven days during the vacation. As a secondary indicator, we used getting into fights during the stay at SB.

As predictors, we considered variables that were likely to attract young people to party package travel rather than to another type of travel. For example, tourists who reported that they had felt attracted to a resort by its party reputation might also select a travel agency advertising party activities. Therefore, an apparent association between travelling with a party package travel and drinking or fighting during the vacation might in fact represent an association between travelling with the intention of partying and the outcome, rather than a causal link between the type of travel and the outcome. We did not include drinking on the vacation as a predictor of outcomes, including fights, as drinking on the vacation would likely be a mediator rather than a confounder of outcomes.

### Ethical considerations

Institutional review boards in Denmark do not review studies unless medication is manipulated, or an invasive procedure is used. We do not believe that our study is in any way in violation of the declaration of Helsinki [13].

In the survey, tourists were informed of the nature and purposes of the study. In doing ethnographic fieldwork, it is not always possible to obtain informed consent from everyone with whom the researcher has contact, but ST who did the fieldwork, openly talked about his work during his stay in SB. During data collection in Bourgas airport, SS and TR wore t-shirts with a logo indicating that they were from the University of Aarhus.

## Results

### Ethnographic fieldwork

SB is located on the Black Sea and attracts a large number of tourists, especially from Northern Europe, with its warm weather, long beach strip, low prices and wild nightlife. Young tourists from Denmark going to SB basically choose between two types of travel agencies: "traditional agencies" that provide airfare and perhaps hotel lodging, sightseeing in the region and one or two evening activities during the week; and "party package agencies" offering flight, hotel and a range of party activities during the week. One of the Danish party package agencies, for instance, had a "Party Power Package" with the following party activities during the week: "Welcome party", "Pub-Crawl", "Nachos Night", "Pool Party", and "Mega Pub Crawl". The party package agencies have trained guides who participate in the festivities day and night. They entertain with risqué shows, singing, dancing, competitions, and drinking games and assist in creating a permissive atmosphere with a clear focus on sex and drunkenness. The guides repeatedly invite tourists to drink heavily and act wildly with verbal instructions such as "bottoms up", "run amok!", "What happens at Sunny Beach stays at Sunny Beach" and so on. Already at the weekly welcome meetings, guides often emphasise that the newly arrived tourists should let go of their inhibitions. As one guide put it during an interview: "We tell them at our meeting to just let go. They should do whatever they want. It's their free choice because they've paid for their vacation. Down here there are no rules. So they should take advantage of that."

It should be added, that most of the Danish tourists that we have interviewed express a strong wish to indulge in wild behaviour. Indeed, as the sociologist Maffesoli has argued, it seems that contemporary western youth have a strong and unbending urge for wildness [14]. The guides assist the tourists in overcoming their inhibitions and let lose. In addition to the use of verbal instructions, guides also purposively make use of loud music to lift the mood, they gather people in big crowds to intensify the festivities, and they encourage people to drink large amounts of alcohol. All this taken together constitutes a powerful constellation that can help tourists in moving far away from the norms and rules of everyday life and into a domain of experimentation and excess. The following field-notes illustrate some of the techniques that the guides employ to make people go wild:

The beer relay race begins after dinner. Three teams are formed, and each participant is supposed to run 15 meters to a waiting guide who holds a large draft beer. The participant must down the beer, run five times around the guide, roll forward, run back and give way to the next in line. Everyone must take two turns, and drink two beers. The participants are overwhelmingly male. The guide says into the microphone: "Now we're gonna play a game we learned in Spain. We're gonna dig holes, so grab a shovel." He takes a break and laughs. There are four teams, each with three players. They are told to dig for 10 minutes. The guide with the microphone tells those not participating to come close and cheer. Some of the other guides encourage others nearby to "come watch, this is cool as hell." The diggers are given small shovels, and they really get to work. Some discuss tactics while digging. Others just give it their all. A female guide runs up to the bar: 'Beer, beer, lots of beer.' She walks around with a tray and serves the diggers, who are laboring under the relentless sun (ST's field notes July 19, 2007).

Thus, the guides actively encourage drinking and they also tend to take part in the drinking – both when they are on duty and when they are not.

On the other hand, the Danish party package agencies arrange meetings for newly arrived tourists where they call attention to some of the main risks in the nightlife of SB. The agencies all have 24-hours services with at least two sober guides who can be called upon in case of emergency. In case of severe accidents, the guides lend first aid, escort victims to the hospital and/or contact the police.

Thus, tourists travelling with Danish party package agencies always have Danish-speaking staff close at hand. Moreover, the party package agencies give wristbands to their customers indicating whom they are travelling with and giving access to an array of privileges such as free entrance to certain discotheques. Tourists who are caught taking illicit drugs have their wristbands taken away, and guides stress that they have a zero-tolerance policy with regard to illicit drugs. Although we have never observed this threat being carried out, the threat is repeated several times to travellers from the moment they arrive in the area.

The party package travel agents cooperate with local bars and discotheques in arranging party activities, and the venues earn considerable amounts of money on the oftentimes hundreds of big spending Danes that the party package agencies bring along. Therefore, venue owners are generally less accepting of security guards' abusive or violent behaviour against guests wearing the wristband. And violent guards are a real problem in SB; i.e. during the summer 2007, a Swedish tourist was beaten to death by local security guards.

### Survey

#### Sample description

On the 13 days, we approached a total of 1238 subjects. A total of 87 women and 197 men refused (26%). Those who indicated that they had time were then informed of the nature of the questionnaire (n = 1068), and among these compliance was 95% (n = 1011). The respondents were 55% male, and the mean age was 19.9 years (range: 13 to 34, standard deviation [SD] = 3.1). The mean number of days spent in Sunny Beach [SB] was 7.8 (range: 1 to 15, SD = 2.2). At total of 18.1% travelled with a long-term partner, 11.9% with their parents, 2.0% with both partner and parents, and 71.6% with neither. Due to missing data, the number of subjects included in each analysis was reduced for particular analyses, as indicated below.

A total of 55 subjects did not report what agency they were travelling with. An approximately equal number of respondents came to SB with a party package travel (n = 450) as a regular package travel agent (n = 506). Subjects going with party package travel agents were more likely to be men (χ^2^(1) = 8.90, p = 0.003), and were younger than those travelling with other package travel agents (mean for party package travellers: 19.2 years, standard deviation [SD] = 1.9; others: 20.6, SD = 3.8, t = -7.5, p < 0.0001). Travellers going with party package travel agents were also much more likely to have been attracted to SB by the place's party reputation (68.0% vs. 32.1%, χ^2^(1) = 121.2, p < 0.0001), and drank more frequently in Denmark (on an ordinal scale from 0 to 8, where 0 is never, and 8 is "5 or more times per week", Mann Whitney U test, Z = 5.12, p < 0.00001).

#### Univariate analyses

We first analyzed whether tourists travelling with companies offering no party activities (n = 236) differed from tourists travelling with one or two weekly party activities (n = 236), and from tourists travelling with agencies with more than two parties a week (n = 388). When comparing those from companies with no party activities with those with one or two party activities, we found no statistically significant difference in drinking 12 or more units (χ^2^(2) = 2.00, p = 0.37). We therefore decided to treat all those travelling with agencies offering fewer than three parties per week as one category.

Among party package travellers, 58.8% drank 12 or more units 6–7 days per week during their stay in SB, 29.4% drank 12 or more units 1–5 days per week, and 11.9% never drank 12 or more units. Among other travellers, 26.5% drank 12 or more units 6–7 days per week during their stay in SB, 29.4% drank 12 or more units 1–5 days per week, and 43.4% never drank 12 or more units. The difference was statistically significant (χ^2^(2) = 126.8, p < 0.0001, N = 860). Very few travellers with any kind of agency used illicit drugs in SB, and the difference was not significant (3.3% of party package travellers vs. 2.1% of travellers with other agencies, χ^2^(1) = 0.73, p = 0.39).

Party package travellers were also more likely to get into fights (12.4% of party package travellers, 5.5% of other travellers, χ^2^(1) = 13.9, p = 0.00019).

The associations with other indicators of alcohol-related harm, including seeking medical treatment (7.8% of party package travellers vs. 5.5% of other travellers, N = 929, χ^2^(1) = 1.98, p = 0.16), seeking medical treatment for an alcohol-related problem (1.6% of party package travellers, 0.9% of other travellers, χ^2^(1) = 1.23, p = 0.27), or for another accident (3.2% of party package travellers, 2.2% of other travellers, χ^2^(1) = 0.83, p = 0.36) were not significant.

Thus, there were strong indications that party package travellers were heavier drinkers than other travellers. However, to test the impact of potential confounders on drinking on the location we next decided to conduct an ordinal regression.

#### Ordinal regression analyses

The ordinal regression analyses were carried out controlling for several confounders, including gender, age, frequency of drinking in Denmark, whether young people were travelling with their parents, travelling with a partner, and whether they reported being attracted to SB by the resort's party reputation.

For drinking 12 or more units per day, both the Spearman rank order correlations and the results of the ordinal regression are shown in table 1.

**Table 1:** Associations between predictors and heavy drinking (n = 760)

	Spearman rank- order correlation	Coefficient	Standard error	z	P > z	Lower 95% Conf. Interval	Higher 95% Conf. Interval
Male gender	***0.43	1.80	0.17	10.31	0.000	1.45	2.14
Age	0.02	-0.03	0.03	-1.07	0.284	-0.08	0.02
Travelling with parents	***-0.43	-2.00	0.30	-6.61	0.000	-2.60	-1.41
Staying +7 days	***-0.30	-0.09	0.20	-0.46	0.648	-0.47	0.29
Attracted by party reputation	***0.19	1.48	0.18	8.22	0.000	1.13	1.83
Frequency of drinking at home	***0.50	0.35	0.06	6.02	0.000	0.24	0.46
Travelling with party package travel	***0.38	0.38	0.19	1.98	0.048	0.00	0.75
Travelling with partner	***-0.41	-1.95	0.24	-8.28	0.000	-2.41	-1.49

The regression analysis for frequency of drinking 12 or more units could be conducted for 757 respondents. The statistics for the analysis indicated that the model added information over an intercept only (likelihood ratio χ^2^(8) = 537.90, p < 0.001, pseudo R^2 ^= 0.33).

Going with a party package travel agent was significantly associated with heavy drinking (coefficient = 0.38, z = 1.98, p = 0.048). Also associated with heavy drinking was male gender (p < 0.001), being attracted to the area by its party reputation (p < 0.001), and frequency of drinking in Denmark (p < 0.001). Travelling with parents (p < 0.001) and a long-term partner (p < 0.001) were both associated with a decreased risk of heavy drinking.

For getting into fights, both the Spearman rank order correlations and the results of the logistic regression are shown in table 2.

**Table 2:** Associations between predictors and getting into a fight: spearman rank order correlations and logistic regression (N = 819)

	Spearman rank- order correlation	Coef.	SE	z	Probability	Lower 95% Conf. Interval	Higher 95% Conf. Interval
Age	-0.05	-0.12	0.06	-1.96	0.050	-0.23	0.00
Male gender	***0.15	0.93	0.30	3.08	0.002	0.34	1.52
Travelling with parents	-0.04	-0.22	0.54	-0.41	0.678	-1.28	0.84
Staying +7 days	-0.05	-0.30	0.38	-0.79	0.430	-1.06	0.45
Attracted by party reputation	**0.10	0.20	0.29	0.67	0.500	-0.37	0.76
Frequency of drinking at home	0.09	0.05	0.09	0.57	0.569	-0.12	0.22
Travelling with party package travel	**0.12	0.30	0.31	0.97	0.332	-0.31	0.91
Travelling with partner	-0.13	-1.23	0.63	-1.95	0.051	-2.45	0.00

The regression analysis for frequency of getting into fights could be conducted for 815 respondents. The statistics for the analysis indicated that the model added information over an intercept only (likelihood ratio χ^2^(8) = 36.15, p < 0.001, pseudo R^2 ^= 0.08).

Going with a party package travel agent was not associated with getting into fights (p = 0.332), but male gender was (p = 0.002). Trends were found for lower risk of getting into fights with higher age (p = 0.050), and travelling with a partner (p = 0.051). Since we did not find that choosing a party package travel agent was associated with getting into fights after controlling for confounders, we did not attempt to analyze whether there was an indirect effect from party package travel to fights mediated through heavier drinking.

## Discussion

The main result of this study was that going with a party package travel agency was associated with higher frequencies of heavy drinking. The majority of young people travelling with a party package agency drank 12 or more units daily or almost daily during their vacation, and the association held after controlling for several known important confounders, including gender, drinking frequency in Denmark, travelling with a partner or parent, and being attracted to SB by the party reputation of the resort. The associations with acute adverse side effects of going with a party package travel on the other hand were modest, and did not hold after controlling for potential confounders.

Those travelling with party package travels differed from those travelling with other types of agencies in terms of their behaviour at home, and it seems very likely that the marketing of party package travels attracts tourists who are looking for a vacation with opportunities for drinking and going out in the nightlife. Thus, whether party package travels causally influence drinking is not established. Our data suggest that they may causally contribute, but that the influence may be small.

## Strengths and limitations

The strengths of this study include an adequate sample size, a high acceptance rate, and the use of on-site ethnographic fieldwork to aid the interpretations of the survey data.

A limitation of this study was the use of cross-sectional data to measure events retrospectively. For instance, respondents' self-reports of their drinking behaviour and their motives for going on the vacation may have been influenced by their experiences during the vacation. Also, their recollection of the actual drinking behaviour may have been biased in a number of ways. Respondents who have consumed more than 12 drinks in a night may have suffered a blackout and failed to remember events, such as being in a fight or having been taken to a doctor.

Also, the amount of data that was collected about each respondent was limited. It was our belief that it would be difficult to engage people who were about to board a plane after a vacation in completing a long questionnaire over several pages.

We did not find any differences between party package travellers and other travellers in terms of adverse outcomes associated with heavy drinking. While we have previously shown that heavy drinking was associated with getting into fights and seeking medical help for an alcohol-related problem, the sensitivity of these items as indicators of heavy drinking is unknown, and potentially low.

Another limitation is that we cannot rule out that pre-existing differences other than those controlled for in the regression analyses were responsible for the differences found in drinking, rather than the party program and behaviour of guides. This limitation will apply to any non-experimental design.

Also, we cannot disentangle the effects of different aspects of the "party package program", for instance pub-crawls, parties, drinking games, foam parties, and the specific weight of the guides' behaviour, and the net effect of being at a party where most of the other guests also are drinking heavily.

## Opportunities for harm reduction?

In terms of preventing drug and alcohol-related harm some interesting questions arise in connection with the role of party package travel agents and their guides. Clearly, the amount of alcohol consumed is higher among the party package travellers. This may be a result of the binge-oriented parties organized by party package agencies. Or it may be because the party package agencies attract youth with a penchant for heavy drinking. Our data give no compelling evidence of higher short-term harm associated with drinking among party package travellers. We have observed on the scene that the guides have high esteem among the young people. They have non-drinking guides with first-help equipment available, and they crack down on illicit drug use. The guides have an ambiguous role: they actively encourage heavy drinking and they try to prevent harm among the tourists.

But does the guides' behaviour function as effective harm reduction to an extent that offsets the negative consequences of the increased alcohol consumption of those travelling with party package travel agents? Do they contribute to the overall very low prevalence of illicit drug use? And is there a potential way of intervening in the context of the SB party scene, to make better use of the "on the spot party guides" as harm reducing agents?

The party scene in SB differs from Denmark in specific ways. It is much more unsafe to walk around alone or in small groups while intoxicated; assaults, robberies and rapes against tourists are reported in high numbers. Certain areas are particularly dangerous to access after dark, such as the beach. When intoxicated young people take taxies at night, drivers often demand extreme prices upon arrival at the destination, harass customers and try to sell illegal drugs. The doormen and security staff can be violent and erratic in their behaviour; the police are not an authority to be trusted. All these factors mean that the things young people do at home to take care of themselves when intoxicated, like taking a taxi rather than walking home or contacting the police when in trouble, are behaviours that could be dangerous in SB [15]. Guides do inform young people of necessary security measures, and simply by being in large groups can protect tourists from many threats.

On the other hand, most of Danish guides are young and also participate in the party scene and for consecutive weeks. Even when the guides are not intoxicated while on duty they are marked by a prolonged period of sleep deprivation and substantial alcohol consumption. In that respect, they may not be the ideal harm reduction advocates. The guides warn tourists against drug use, but they also have a clear tendency to downplay the many dangers of heavy drinking.

Another strategy to prevent harm would be to prohibit the sale of party package travels to young people under the legal drinking age. In Denmark the legal age for drinking in bars and restaurants is 18 years, and it would seem consistent to prohibit selling pub-crawls with alcohol to young people aged less than 18 years for instance. As always, potential harm reduction should be weighted against the potential benefits of having guides available for the young people who do drink while on vacation. In general, guides could do more to warn against the negative effects of heavy drinking and give advice about how to limit some of these negative effects – for instance by emphasising that it is vital to eat food and drink sufficient amounts of water. Guides should also avoid encouraging heavy drinking and instead shift their focus to other party activities such as dancing and non-alcoholic party games and competitions.

Obviously, travel agencies are not interested in over-stating the dangers at travel destinations or in appearing to be preaching from a "moral high ground", but with the right dialogue between authorities and agencies, agencies could be motivated to make a stronger effort. This dialogue should also involve the local Bulgarian authorities, who could do a much better job in reducing the alarming crime rates in the Bulgarian nightlife resorts.

## Conclusion

Young people going with party package travel agencies drink more than visitors travelling with other travel package agencies, but the difference is small when controlling for other confounders. The behaviours of guides constitute a mixture of risk-inducing encouragement of drinking and harm reduction promotion. The situation at holiday resorts calls for harm reduction measures.

## Competing interests

The authors declare that they have no competing interests.

## Authors' contributions

ST and MH planned the quantitative study and adopted the questionnaire from Mark Bellis' and Karen Hughes' original questionnaire. ST planned the ethnographic study. ST, AS, and TR collected the questionnaires and carried out the ethnographic observations. MH drafted the manuscript and carried out the statistical analyses. ST drafted the sections about the ethnographic data, and all authors read and discussed the article and approved the final manuscript.

## Pre-publication history

The pre-publication history for this paper can be accessed here:


